# The potential mechanism of treating IC/BPS with hyperbaric oxygen by reducing vascular endothelial growth inhibitor and hypoxia-inducible factor-1α

**DOI:** 10.55730/1300-0144.5762

**Published:** 2023-12-11

**Authors:** Jing ZHANG, Jiarui KANG, Liyang WU

**Affiliations:** 1 Department of Hyperbaric Oxygen, Capital Medical University, Beijing Chao-Yang Hospital, Beijing, China; 2 Department of Pathology, Fourth Medical Centre of Chinese People’s Liberation Army General Hospital, Beijing, China; 3 Department of Urology, Capital Medical University, Beijing Chao-Yang Hospital, Beijing, China

**Keywords:** Interstitial cystitis/bladder pain syndrome, hyperbaric oxygen, vascular endothelial growth inhibitor, hypoxia-inducible factor-1α

## Abstract

**Background/aim:**

To investigate the roles of vascular endothelial growth inhibitor (VEGI) and hypoxia-inducible factor-1α (HIF-1α) in the treatment of refractory interstitial cystitis/bladder pain syndrome (IC/BPS) with hyperbaric oxygen (HBO).

**Materials and methods:**

A total of 38 patients were included. They were assessed before and 6 months after HBO treatment. Three-day voiding diaries were recorded, and O’leary-Sant scores, visual analog scale (VAS) scores, quality of life (QoL) scores, pelvic pain, and urgency/frequency (PUF) scores were evaluated. Bladder capacity was assessed by cystoscopy. Bladder mucosa was collected for Western blot, qRT-PCR, and immunofluorescence staining to compare the expression of VEGI and HIF-1α before and after treatment.

**Results:**

Compared with before treatment, patients showed significant improvements in 24-h voiding frequency (15.32 ± 5.38 times), nocturia (3.71 ± 1.80 times), O’leary-Sant score (20.45 ± 5.62 points), VAS score (41.76 ± 17.88 points), QoL score (3.03 ± 1.44 points), and PUF score (19.95 ± 6.46 points) after treatment (p < 0.05). There was no significant difference in bladder capacity before and after treatment (p ≥ 0.05). The expression levels of VEGI and HIF-1α protein and mRNA were significantly decreased 6 months after treatment compared with before treatment. Immunofluorescence staining results showed that the double positive expression of VEGI and HIF-1α protein in bladder tissue of IC/BPS patients after HBO treatment quantitatively decreased significantly.

**Conclusion:**

This study identified a possible mechanism by which VEGI and HIF-1α expression decreased after HBO treatment due to hypoxia reversal, which improved symptoms in IC/BPS patients.

## 1. Introduction

Interstitial cystitis (IC)/bladder pain syndrome (BPS) is a disease characterized by primary, chronic, and sterile inflammation of the bladder, which causes discomfort or chronic pain in the bladder area and pelvic region, and is associated with symptoms such as urinary frequency and urgency, severely affecting the physiological and psychological status of patients [1].

Currently, traditional treatments (including oral medications, bladder instillation, and hydrodistension) provide only short-term symptom relief for IC/BPS and do not prevent bladder fibrosis and contracture. However, microvascular improvement appears to play an important role in preventing the fibrotic process of tissue and organs. Previous studies have reported reduced bladder blood flow in IC/BPS patients, particularly during bladder filling [2], suggesting that the pathological and physiological changes observed in IC/BPS may be due to bladder tissue ischemia/hypoxia[3].

In recent years, literature has reported that hyperbaric oxygen (HBO) therapy has significantly alleviated refractory IC/BPS that does not respond to traditional treatments and maintains long-term effects [4–6]. Unlike traditional treatments, HBO increases the oxygen concentration in hypoxic tissue, enhancing the ability to regenerate and repair local microvasculature. This opens up new and effective treatment concepts, pathways, and methods for IC/BPS and provides new directions.

Oxygen free radicals produced under hypoxic or anoxic conditions promote the induction of vascular endothelial growth inhibitor (VEGI), which induces apoptosis of endothelial cells and inhibits the formation of tissue microvasculature [7,8]. On the other hand, it remains to be further studied whether the effect of VEGI is inhibited due to the improvement of hypoxia under hyperbaric oxygen supply, leading to a shift of the balance towards proangiogenic factors and away from antiangiogenic factors.

As far as we know, there are no reports on the correlation between VEGI expression in the bladder of IC/BPS patients and the severity of angiogenesis and hypoxia. Lee et al. have described the increased expression of hypoxia-inducible factor-1α (HIF-1α) and its correlation with the formation of glomerular-like bleeding in patients with interstitial cystitis [9]. Meanwhile, glomerulations result from petechial bleeding caused by bladder hydrodistention, higher pressures, and ischemia [2]. This study aims to investigate the potential relationship between VEGI, HIF-1α, and clinical symptoms of IC/BPS in patients undergoing HBO therapy.

## 2. Materials and methods

### 2.1.Patients and sample collection

Bladder specimens were obtained from 38 IC/BPS patients (2 males, and 36 females) who underwent cystoscopy under anesthesia with hydrodistension (Table 1). All patients met the diagnostic criteria of the 2015 AUA guidelines [10], with ages ranging from 25 to 77 years (mean age, 53.0 ± 13.3 years). All patients had received medications (including oral medicine: antihistamines, amitriptyline, pentosane polysulphate, and immunosuppressants, intravesical therapy: lidocaine, heparin, hyaluronic acid and chondroitin sulphate, or combination therapy) with poor efficacy. This study was approved by the ethics committee of Beijing Chao-Yang Hospital affiliated with Capital Medical University (2018-ke-54, February 12, 2018), and all patients provided written informed consent and signed an HBO treatment informed consent form after evaluation by an HBO physician.

### 2.2. All patients underwent hydrodistension under cystoscopy and a random bladder mucosal biopsy before and 6 months after HBO treatment

The procedure was performed using a rigid cystoscope (18Fr) under general venous anesthesia, as follows: the bladder perfusion pressure was maintained at 80 cmH_2_O for 5 min, after which the fluid was drained from the bladder. The cystoscope was then reinserted to examine the mucosa of the bladder wall and a positive sign of erythema was defined as more than 3 consecutive cystoscopic fields with over 10 bleeding points each. All 38 IC/BPS patients showed positive signs of erythema and 15 of them with hunner’s ulcer under the cystoscopy (Table 1).

Bladder specimens were collected with random biopsy after hydrodistension, with sampling sites including the left, right, and posterior walls of the bladder. All specimens were divided into 2 parts, with half of the specimen stored at −80 °C and the other half stored in formalin. We randomly selected 9 cases for specimen collection, with samples taken both before and 6 months after HBO treatment. These specimens underwent various tests, including pathological examination, qRT-PCR, Western blot, and immunofluorescence staining for VEGI and HIF-1α.

### 2.3. HBO treatment

We conducted two courses of HBO treatment for IC/BPS patients. During the treatment, patients breathed 100% oxygen through a mask in a hyperbaric oxygen chamber at 2 ATA for 120 min per session, including compression and decompression time, with 60 min of oxygen inhalation. Each course consisted of 20 treatments, given five times a week, with a one-week break before starting the second course.

### 2.4. Observation results

The severity of symptoms was evaluated before and 6 months after the end of HBO treatment in IC/BPS patients using a three-day voiding diary, O’Leary-Sant score, Visual Analogue Scale (VAS), Quality of Life (QoL) index, and Pelvic Pain, Urgency and Frequency (PUF) score.

### 2.5. qRT-PCR

Total RNA was extracted from bladder samples by homogenization in TRizol reagent (Invitrogen, Carlsbad, CA) and subsequently used for cDNA synthesis using a kit (Thermo, #K1622). Quadruplicates from 4 independent experiments were analyzed by quantitative real-time polymerase chain reaction (qPCR) on the 7500 real-time PCR system (ROCHE, LC480). The conditions were initial denaturation at 95 °C for 10 min, followed by 40 amplification cycles with denaturation at 95 °C for 15 s and annealing and extension at 60 °C for 60 s. Each experiment was done three times, and the average values were used for analysis. β-actin was internal reference. Ct = −1/lg(1+Ex)*lgX_0_+lgN/lg(1+Ex).

### 2.6. Western blot

Protein concentration in cell lysates were determined using the DC Protein Assay kit (Bio-Rad) and an ELx800 spectrophotometer (Bio-TekTM). Equal amounts of proteins were separated by sodium dodecyl sulfate-polyacrylamide gel electrophoresis (SDS-PAGE) and blotted onto nitrocellulose sheets. Proteins were then probed with the VEGI(sc-53975, Santa Cruz, Santa Cruz, CA; dilution 1:1500) or HIF-1α(#36169, Cell Signaling, Beverly, MA; dilution 1:500) antibodies and peroxidase-conjugated secondary antibodies. Protein bands were visualized using the SupersignalTM West Dura system (Pierce Biotechnology, Inc., Rockford, IL) and photographed using a UVITech imager (UVITech, Inc., Cambridge, UK).

### 2.7. Immunofluorescence staining

Slides were washed in PBS and incubated sequentially for 30 min with goat serum, overnight at 4 °C with primary antibodies (VEGI, sc-53975, Santa Cruz, Santa Cruz, CA; dilution 1:1500) and for 1 h at 37 °C with Cy3 labeling sheep anti-mouse IgG (1:50). The slides were then washed in PBS and incubated with goat serum. The primary antibody (HIF-1α, #36169, Cell Signaling, Beverly, MA; dilution 1:1000) was used overnight at 4 °C, and FITC labeling sheep anti-rabbit IgG (1:50) for 1 h at 37 °C. DAPI was added before PBS. Images were collected using a fluorescence microscope (Olympus, BX53).

### 2.8. Statistical analysis

**Figure 1:**
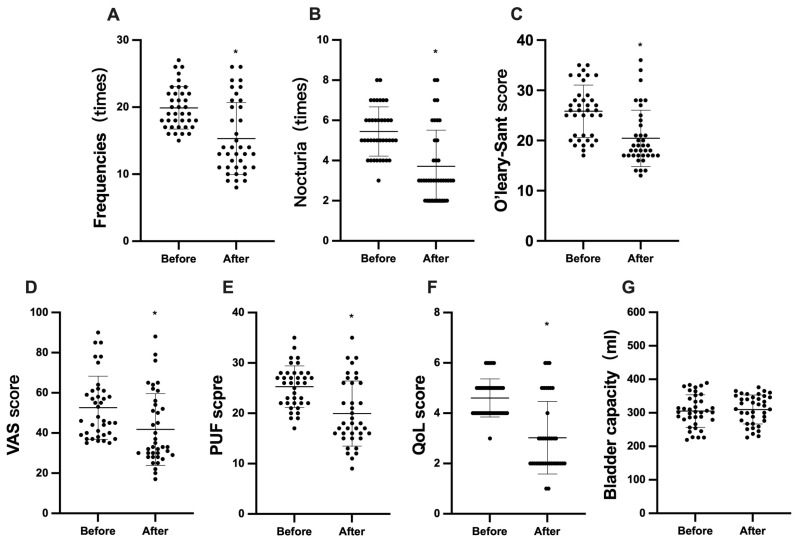
Comparisons of average 24-h frequency (A), nocturia (B), O’leary-Sant score (C), VAS score (D), PUF score (E), QoL score (F), and bladder capacity (G) before and after HBO treatment. * a significant difference (p < 0.05). VAS, visual analog scale; PUF, pelvic pain and urgency/frequency; QoL, quality of life;

Statistical analysis was performed with SPSS software, version 22.0 (SPSS Inc, Chicago, IL). All values are reported as Mean ± SD. 2^-ΔΔCT values were used for comparative analysis between groups in qRT-PCR. Comparisons of the means observed in different groups were performed using the paired t-test, and p < 0.05 was set as statistically significant. GraphPad Prism 9 (GraphPad Software, La Jolla, CA) was used to draw Figure 1.

## 3. Results

In 38 patients, the frequency of urination within 24 h (Figure 1A) decreased significantly from 19.89 ± 3.16 times before HBO therapy to 15.32 ± 5.38 times after treatment (p < 0.05). The nocturia (Figure 1B) also decreased from 5.45 ± 1.22 times before treatment to 3.71 ± 1.80 times after treatment (p < 0.05). After HBO therapy, the O’leary-Sant score (Figure 1C) decreased from a baseline of 25.84 ± 5.22 to 20.45 ± 5.62 (p < 0.05), the VAS score (Figure 1D) decreased from 52.61 ± 15.69 to 41.76 ± 17.88 (p < 0.05), the PUF score (Figure 1E) decreased from 25.29 ± 4.13 before treatment to 19.95 ± 6.46 after treatment (p < 0.05), and the QoL score (Figure 1F) decreased from 4.61 ± 0.75 to 3.03 ± 1.44 (p < 0.05).

After 6 months of HBO therapy, all patients underwent cystoscopy again. There was no significant difference in bladder capacity under anesthesia between treatment before (305.40 ± 48.60 mL) and after (309.80 ± 43.25 mL) treatment (p ≥ 0.05) (Figure 1G). From the microscopic presentation, we found that the number of patients with hunner’s ulcer decreased from 15 cases before treatment to 13 cases after treatment. In addition, some patients could observe a relative decrease in the number of ulcers, although there was no disappearance of ulcers; and the number of patients with positive erythema also decreased from 38 cases before treatment to 31 cases after treatment (Table 2).

Comprehensive therapy is mandatory in refractory IC/BPS. Eighteen patients received oral medicine, 5 patients received intravesical therapy, 11 patients received oral medicine and intravesical therapy, and 4 other patients did not receive medication during HBO therapy (Table 3).

**Figure 2:**
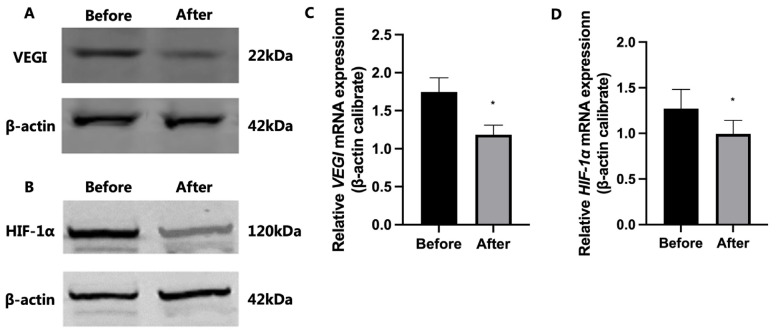
Typical pictures of the expression of VEGI and HIF-1α protein (A and B) and mRNA (C and D) in IC/BPS patients before and after HBO treatment. * a significant difference (p < 0.05).

Western blot analysis demonstrated that the expression levels of VEGI and HIF-1α proteins in bladder tissue of IC/BPS patients after HBO treatment were significantly lower than those before HBO treatment (Figures 2A and 2B). qRT-PCR also produced similar results, with the expression of VEGI and HIF-1α mRNA being significantly decreased in the bladder tissue of patients after HBO treatment compared to before (p < 0.05; Figures 2C and 2D).

**Figure 3:**
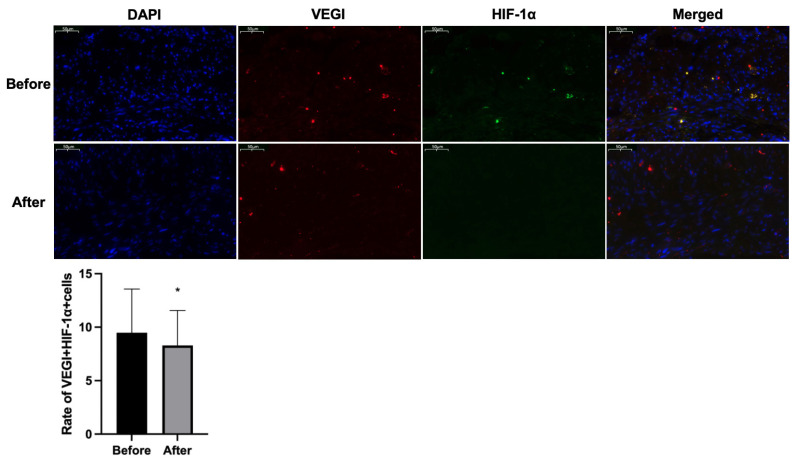
Typical pictures of the expression of VEGI and HIF-1α in bladder wall of IC/BPS patients before and after HBO treatment. * a significant difference (p < 0.05).

Immunofluorescence results showed a significant reduction in the expression of VEGI and HIF-1α in the bladder tissue of IC/BPS patients after HBO treatment compared to before. Using ImageJ software, the number of total cells and the number of VEGI and HIF-1α double-positive expression cells were counted in the field of view. Compared with bladder tissue before HBO treatment, the double-positive expression of VEGI and HIF-1α protein in bladder tissue of IC/BPS patients after HBO treatment quantitatively decreased significantly (Figure 3).

## 4. Disscussion

The characteristics of IC/BPS are the destruction of the urethral barrier and the loss of its normal function. The damaged urethral barrier loses its normal impermeability to urine solutes, leading to bladder pain, urgency, and frequency. Lee et al. reported that a decrease in bladder infusion volume in IC/BPS patients can lead to bladder tissue hypoxia or ischemia [9]. Hypoxia can alter the expression of tight junction proteins, increase the permeability of the bladder urethral epithelium, allow small ions to pass through the blood-urine barrier of the bladder [11], and induce related symptoms. However, the reversal of hypoxia seems to improve urinary symptoms. From our results, it can be seen that after receiving HBO treatment, patients’ urinary symptoms were improved, and there was no significant decrease in bladder capacity under anesthesia compared with the presurgical period, which confirmed the efficacy of HBO. It is well known that the bladder of IC/BPS patients progresses slowly towards fibrosis, either due to repeated inflammatory reactions or weakening of nerve nourishment. HBO can, on the one hand, delay bladder fibrosis and stop the reduction of bladder capacity; on the other hand, it can repair the bladder microvessels and mucosal layer and improve inflammation to relieve voiding symptoms.

The basic principle of HBO treatment is to inhale pure oxygen in an environment exceeding one atmosphere, which increases the oxygen saturation in circulation and the oxygen diffusion gradient between surrounding tissues, causing a sharp increase in oxygen concentration in damaged hypoxic tissues, thus enhancing local vascular regeneration and repair [12]. Unlike traditional IC/BPS treatment, HBO treatment for IC/BPS is based on the repair and regeneration of bladder tissue blood vessels, further promoting damaged tissue growth and fundamentally restoring damaged bladder mucosa, avoiding extensive fibrosis and contracture of the bladder. This seems to be the reason why HBO is still effective for refractory IC/BPS patients who are not responsive to traditional treatments, and why the Canadian Urological Association has included it in the treatment guidelines for IC/BPS[4].

The local microvascular nutrition and support of the bladder are the basis for the integrity of the mucosa, and the restoration of integrity inevitably requires the supply of neovascularization. From the treatment mechanism of HBO, it can be seen that the repair of damaged microvessels is a prerequisite for normal blood supply to the bladder mucosa. However, HBO only provides an external environment, and the real effect is the internal factor, namely the joint action of proangiogenic factors and antiangiogenic factors in improving hypoxia.

Therefore, we explored the possible relationship between the internal factors- VEGI and HIF-1α. VEGI is a natural microvascular growth inhibitor widely present in endothelial cells. Under low oxygen or hypoxic conditions, oxygen free radicals promote VEGI-induced endothelial cell apoptosis, thereby inhibiting tissue microvascular formation [7,8]. HIF-1α is a key transcription factor in the angiogenesis of the hypoxic response [13,14], helping cells adapt to hypoxia by enhancing cell survival and proliferation [14,15]. Our results showed that the protein and mRNA expression of VEGI and HIF-1α were significantly reduced after HBO therapy compared to before treatment, confirming the hypothesis mentioned above.

High expression of VEGI induces apoptosis of endothelial cells and inhibits microvascular formation, which may be aggravated in low oxygen or hypoxic conditions [8]. Conversely, under HBO supply, the improvement of hypoxia or lack of oxygen down-regulates HIF-1α, thereby inhibiting the effect of VEGI, tilting the balance of proangiogenic and antiangiogenic factors towards proangiogenic factors, and ultimately improving the symptoms of patients and delaying the fibrosis process of bladder.

The main cause of fibrosis and contracture of the bladder in refractory IC/BPS patients is due to bladder interstitial inflammation caused by bladder mucosal dysfunction, which subsequently leads to local microvascular damage, decreased mucosal repair capacity, and inadequate local tissue blood supply [16]. Therefore, repairing and regenerating microvessels may improve local blood supply to the bladder and repair bladder mucosa. The improvement of symptoms in IC/BPS patients after HBO therapy and no further reduction in bladder capacity confirmed this view. At present, the treatment of IC/BPS, such as antihistamines, antiinflammatory drugs, or glucosamine sulfate layer repair therapy, cannot repair and regenerate the local microvessels of the bladder and restore the blood supply of bladder. However, HBO can provide a guarantee for the long-term prognosis of the disease by inducing microvascular repair and regeneration through the improvement of hypoxia.

There are still some limitations in this study. We found that HBO was not effective for all IC/BPS patients, and the protein expression of VEGI and HIF-1α should be compared between effective and ineffective patients to clarify their decisive significance in HBO treatment of IC/BPS. The reduced VEGI and HIF-1α expressions are only indirect pieces of evidence of vessel regeneration. The direct evidence of increased microvascular after HBO therapy is needed. As for IC/BPS, comprehensive treatment is still the most commonly used method, and there is further room for exploration regarding the differences in therapeutic effects and protein expression obtained by different treatment methods.

## Figures and Tables

**Table 1:** Characteristics of patients.

Characteristics	
Age (years)	53.0 ± 13.3
Sex (male: female)	2: 36
Erythema: positive: negative	38: 0
Ulcerative: non-ulcerative	15: 23

**Table 2:** Comparison of cystoscopic performance before and after HBO treatment.

Characteristics	Before	After
Ulcerative (cases)	15	13
Erythema: positive (cases)	38	31

**Table 3:** Medication during the HBO therapy.

Medication	Number of patients (cases)
Oral medicine (antihistamines, amitriptyline, pentosane polysulphate, immunosuppressants)	18
Intravesical therapy (lidocaine, heparin, hyaluronic acid and chondroitin sulphate)	5
Combination therapy (oral medicine + intravesical therapy)	11
None	4

## References

[1] Bendrick TR Sitenga GL Booth C Sacco MP Erie C The implications of mental health and trauma in interstitial cystitis Health Psychology Research 2022 10 4 40321 https://doi:10.52965/001c.40321 36425233 10.52965/001c.40321 PMC9680853

[2] Tamaki M Saito R Ogawa O Yoshimura N Ueda T Possible mechanisms inducing glomerulations in interstitial cystitis: relationship between endoscopic findings and expression of angiogenic growth factors Journal of Urology 2004 172 3 945 948 https://doi:10.1097/01.ju.0000135009.55905.cb 15311005 10.1097/01.ju.0000135009.55905.cb

[3] Jiang YH Jhang JF Ho HC Chiou DY Kuo HC Urine oxidative stress biomarkers as novel biomarkers in interstitial cystitis/bladder pain syndrome Biomedicines 2022 10 7 1701 https://doi:10.3390/biomedicines10071701 35885006 10.3390/biomedicines10071701 PMC9312927

[4] Cox A Golda N Nadeau G Curtis NJ Carr L CUA guideline: Diagnosis and treatment of interstitial cystitis/bladder pain syndrome Canadian Urological Association Journal 2016 10 5–6 E136 E155 https://doi:10.5489/cuaj.3786 27790294 10.5489/cuaj.3786 PMC5065402

[5] Minami A Tanaka T Otoshi T Kuratsukuri K Nakatani T Hyperbaric oxygen significantly improves frequent urination, hyperalgesia, and tissue damage in a mouse long-lasting cystitis model induced by an intravesical instillation of hydrogen peroxide Neurourology and Urodynamics 2019 38 1 97 106 https://doi:10.1002/nau.23822 30411813 10.1002/nau.23822

[6] Tanaka T Nitta Y Morimoto K Nishikawa N Nishihara C Hyperbaric oxygen therapy for painful bladder syndrome/interstitial cystitis resistant to conventional treatments: long-term results of a case series in Japan BMC Urology 2011 11 1 11 https://doi:10.1186/1471-2490-11-11 21609485 10.1186/1471-2490-11-11 PMC3116481

[7] Zhao Q Kun D Hong B Deng X Guo S Identification of novel proteins interacting with vascular endothelial growth inhibitor 174 in renal cell carcinoma Anticancer Research 2017 37 8 4379 4388 https://doi:10.21873/anticanres.11832 28739731 10.21873/anticanres.11832

[8] Jiang F Chen Q Huang L Wang Y Zhang Z TNFSF15 inhibits blood retinal barrier breakdown induced by diabetes International Journal of Molecular Sciences 2016 17 5 615 https://doi:10.3390/ijms17050615 27120595 10.3390/ijms17050615 PMC4881442

[9] Lee JD Lee MH Increased expression of hypoxia-inducible factor-1α and vascular endothelial growth factor associated with glomerulation formation in patients with interstitial cystitis Urology 2011 78 4 971.e11 e15 https://doi:10.1016/j.urology.2011.05.050 10.1016/j.urology.2011.05.050 21813166

[10] Hanno PM Erickson D Moldwin R Faraday MM Diagnosis and treatment of interstitial cystitis/bladder pain syndrome: AUA guideline amendment Journal of Urology 2015 193 5 1545 1553 https://doi:10.1016/j.juro.2015.01.086 25623737 10.1016/j.juro.2015.01.086

[11] Eldrup J Thorup J Nielsen SL Hald T Hainau B Permeability and ultrastructure of human bladder epithelium British Journal of Urology 1983 55 5 488 492 https://doi:10.1111/j.1464-410x.1983.tb03354.x 6626894 10.1111/j.1464-410x.1983.tb03354.x

[12] Chen C Chen W Li Y Dong Y Teng X Hyperbaric oxygen protects against myocardial reperfusion injury via the inhibition of inflammation and the modulation of autophagy Oncotarget 2017 8 67 111522 111534 https://doi:10.18632/oncotarget.22869 29340072 10.18632/oncotarget.22869 PMC5762340

[13] Tang CH Hwang LY Lee TH Chloride channel ClC-3 in gills of the euryhaline teleost, Tetraodon nigroviridis: expression, localization and the possible role of chloride absorption The Journal of Experimental Biology 2010 213 5 683 693 https://doi:10.1242/jeb.040212 20154183 10.1242/jeb.040212 PMC4074241

[14] Dell JR Mokrzycki ML Jayne CJ Differentiating interstitial cystitis from similar conditions commonly seen in gynecologic practice European Journal of Obstetrics & Gynecology and Reproductive Biology 2009 144 2 105 109 https://doi:10.1016/j.ejogrb.2009.02.050 19409685 10.1016/j.ejogrb.2009.02.050

[15] Parsons CL The role of the urinary epithelium in the pathogenesis of interstitial cystitis/prostatitis/urethritis Urology 2007 69 4 9 16 https://doi:10.1016/j.urology.2006.03.084 17462486 10.1016/j.urology.2006.03.084

[16] Liu M Shen S Kendig DM Mahavadi S Murthy KS Inhibition of NMDAR reduces bladder hypertrophy and improves bladder function in cyclophosphamide induced cystitis Journal of Urology 2015 193 5 1676 1683 https://doi:10.1016/j.juro.2014.12.092 25572034 10.1016/j.juro.2014.12.092 PMC4861050

